# *In vitro* activity of cefiderocol against comparators (ceftazidime-avibactam, ceftazidime-avibactam/ aztreonam combination, and colistin) against clinical isolates of meropenem-resistant *Klebsiella pneumoniae* from India

**DOI:** 10.1128/spectrum.00847-23

**Published:** 2023-09-22

**Authors:** Kalyani Borde, M. A. Kareem, Ratna Mani Sharma, S. Manick Dass, Vedantham Ravi, Dilip Mathai

**Affiliations:** 1 Department of Microbiology, Apollo Institute of Medical Sciences and Research, Hyderabad, India; 2 Department of Internal Medicine and Adult Infectious Diseases, Apollo Institute of Medical Sciences and Research, Hyderabad, India; University of Cincinnati, Cincinnati, Ohio, USA

**Keywords:** cefiderocol, carbapenem resistance, *Klebsiella pneumoniae*

## Abstract

Cefiderocol (FDC), a novel siderophore drug, is active against Gram-negative bacteria producing carbapenemases, including metallo-beta-lactamases. The objective of this study is to compare the *in vitro* activity of FDC with ceftazidime-avibactam (CZA), CZA/aztreonam (AT) combination, and colistin (CST), in clinical isolates of meropenem-resistant (MER-R) *Klebsiella pneumoniae*. From the 2,052 clinical specimens submitted for culture testing, 245 *K*. *pneumoniae* isolates were recovered within a 6-month period in 2021. One hundred three non-duplicate, non-outbreak, MER-R (minimum inhibitory concentration, MIC >4 µg/mL) strains were included in the study. Identification and susceptibility were performed using VITEK-2 (bioMérieux). Meropenem-susceptible isolates (*n* = 10) served as controls. For FDC, broth microdilution (BMD) was performed after in-house standardization. Disk diffusion (Liofilchem, Italy) and broth microdilution (ComASP, STC, Liofilchem, Italy) were used for susceptibility testing of CZA and CST, respectively. Synergy testing for CZA and AT was performed using disk approximation method. CLSI breakpoints were used for the interpretation of the results. For FDC, MIC_50_ and MIC_90_ were 2 and 8 µg/mL, respectively. A total of 80% of isolates were susceptible to FDC, 26.2% of isolates were susceptible to CZA, synergy testing with CZA/AT was positive for 74 (72%) of the isolates, and 89.3% were intermediate to CST. Nine (8.7%) were susceptible only to FDC. FDC is active *in vitro* against MER-R *K. pneumoniae* >CZA/AT > CZA > CST*,* as observed in this study, applying CLSI criteria. Clinico-microbiological studies should be performed to assess the clinical efficacy of this novel drug in this region with a high prevalence of carbapenem resistance among Gram-negative organisms.

**Importance:**

Management of infections with multi-drug resistant *Klebsiella pneumoniae* is a major challenge in hospital settings, with few treatment options. In this study, the authors aim to assess the *in vitro* susceptibility of these clinical isolates to cefiderocol, a novel siderophore. Comparators are colistin, ceftazidime-avibactam, and ceftazidime-avibactam/aztreonam synergy, which are currently available options for treatment in this region. Baseline-resistance rates against cefiderocol are higher than those in the previously published studies, with MIC50 and MIC90 at 2 and 8 µg/mL, respectively.

## INTRODUCTION

Cefiderocol (FDC) is a novel siderophore, with an ingenious description of its mechanism of action as “Trojan horse.” The drug enters the bacterial cell via active iron transporters, which help overcome beta-lactamase-mediated resistance in Gram-negative organisms (1). The Infectious Disease Society of America and the European Society for Clinical Microbiologists and Infectious Diseases have recommended this drug for treating carbapenem-resistant Enterobacterales (CRE) (2, 3). Various large-scale trials have demonstrated high-level *in vitro* activity of this drug against carbapenem-resistant organisms (4, 5). However, the data from countries such as India, with high burden of carbapenem resistance, particularly metallo-beta-lactamase (MBL)-mediated resistance, are lacking. According to the Indian national surveillance data, carbapenem resistance is high among clinical isolates of *Klebsiella pneumoniae* (6). The aim of this study was to evaluate the *in vitro* activity of FDC, a bactericidal agent, and compare it with currently available bactericidal therapy options such as ceftazidime-avibactam (CZA) and colistin (CST).

## MATERIALS AND METHODS

### Place of study

The study was carried out prospectively at the microbiology laboratories attached to a teaching hospital and a tertiary care center in southern India, during 2021. The study was approved by the institutional research and ethical committee (AIMSR/IRB/2020/11/B/7).

### Isolate selection

From the clinical samples submitted for bacterial cultures, 113 non-repetitive isolates of *K. pneumoniae* as identified by VITEK-2 (bioMérieux) were selected. Meropenem susceptibility was performed during routine laboratory testing using VITEK-2 GN cards, as per local protocols. Meropenem-resistant isolates [defined as isolates with minimum inhibitory concentration (MIC) of ≥4 µg/mL] were included in the study. Other antibiotics tested with VITEK-2 were amikacin, ampicillin, amoxicillin-clavulanic acid, cefuroxime, ceftriaxone, cefepime, ciprofloxacin, cotrimoxazole, gentamicin, imipenem, nitrofurantoin, piperacillin-tazobactam, tigecycline, and CST. Isolates were stored at −80°C in glycerol broth, until further testing.

### MIC testing

**Fig 1:**
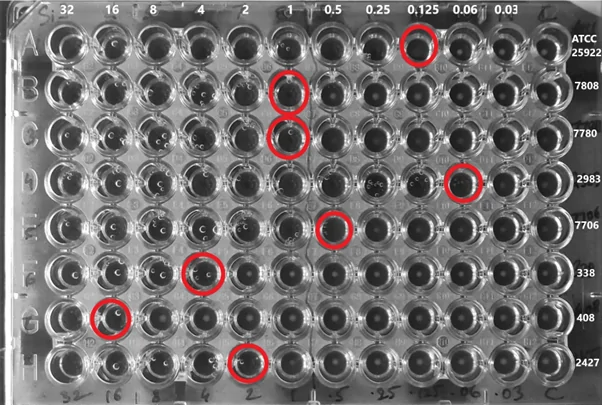
Reading of cefiderocol broth microdilution testing.

FDC drug in pure form (research use only) was obtained from Shionogi & Co. Ltd. Dilutions were prepared in round bottom microtiter plates, ranging from 0.03 µg/mL to 32 µg/mL. This range was selected to cover the MIC ranges of quality control strains at the lower end. The plates were stored at −80°C. When sufficient isolates were accumulated, plates were thawed and inoculated. For preparing inoculum, research use only iron-deficient, cation adjusted Mueller-Hinton broth (ID-CAMHB; Thermo Fisher, USA) was used. BMD plates were incubated at 35 ± 2°C for 16–20 hours. Results were interpreted as the first well where visible bacterial growth was inhibited (Fig. 1). In case of trailing growth (tiny button or light or faint growth as compared to the growth control well), the MIC was read as the first well where the growth was significantly reduced (7). All 113 isolates were tested for FDC MIC using BMD. CST MIC testing was performed for all 113 isolates using commercial lyophilized broth microdilution plates (Liofilchem, Italy) for the range of 0.25–16 µg/mL. CLSI guidelines (M100, 32nd edition) were used for interpretation (7).

### Disk diffusion testing

**Fig 2:**
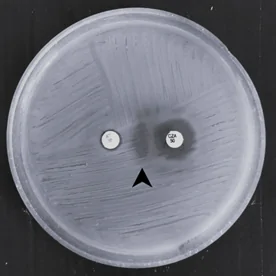
Synergy testing between aztreonam and ceftazidime-avibactam by disk diffusion method. Arrowhead: zone distortion showing synergy between aztreonam and ceftazidime-avibactam.

FDC (30 µg), CZA (30/20 µg), and aztreonam (30 µg) disks (Liofilchem, Italy) were tested as per Kirby-Bauer disk diffusion method on Mueller-Hinton agar (HiMedia Laboratories, India) as per CLSI guidelines (7). Zone diameters were read after incubation at 35 ± 2°C for 16–18 h. FDC disk diffusion was performed for 52 isolates as the disks were received at a later date due to COVID-related delay in shipments. The synergy between CZA and AT was tested for all 103 isolates by observing zone distortion (Fig. 2) between the disks of CZA and AT placed 20 mm apart, as in disk approximation methods (8, 9).

### Quality control

For MIC as well as disk diffusion testing, quality control strains were selected as per the CLSI guidelines (7). For susceptibility testing of FDC, American Type Culture Collection (ATCC) strains of *E. coli* ATCC 25922 and *Pseudomonas aeruginosa* ATCC 27853 were used. For CST susceptibility testing, *E. coli* ATCC 25922 and *E. coli* National Collection of Type Cultures 13486 were used.

### Agreement analysis

Taking BMD as the gold standard, categorical agreement (CA) with the disk diffusion was calculated. Very major errors (VME), major errors (ME), and minor errors (mE) were also calculated. VME is expressed as the percentage of results where resistant (R) is categorized as susceptible (S). ME is expressed as the percentage of results where susceptible (S) is categorized as resistant (R), and that for mE is when susceptible (S) or resistant (R) results are categorized as intermediate (I).

## RESULTS

Of the 113 clinical isolates of *K. pneumoniae*, 37%, 22%, 20%, 14%, and 7% of the isolates were from hospitalized patients with blood, urine, respiratory tract, pus, and from other sites, respectively.

### FDC susceptibility testing

**Fig 3:**
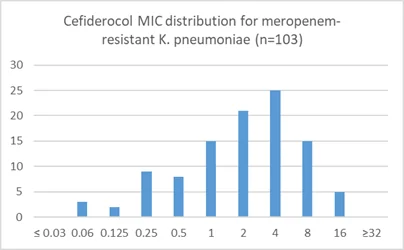
Distribution of minimum inhibitory concentrations for cefiderocol for meropenem-resistant *Klebsiella pneumoniae* isolates included in the study.

FDC MICs for meropenem-susceptible isolates (*n* = 10) ranged from ≤0.03 to 2 µg/mL. For meropenem-resistant isolates (*n* = 103), MIC_50_ and MIC_90_ were 2 and 8 µg/mL, respectively. Using BMD, 80.6% (83/103) isolates were susceptible to FDC (MIC ≤ 4 µg/mL), 15 (14.5%) were intermediate (MIC = 8 µg/mL), and 5 (4.8%) showed resistance (MIC ≥ 16 µg/mL), according to the CLSI breakpoints (Fig. 3).

FDC disk diffusion was performed for 52 of these 103 isolates (Table 1). With the CLSI breakpoints, categorical agreement with BMD was 90%, 50%, and 100% for susceptible, intermediate, and resistant categories, respectively. ME occurred in 1.9% and mE in 9.6%. There were no VME.

**TABLE 1:** Relative distribution of zone diameters and minimum inhibitory concentrations for cefiderocol for meropenem-resistant *Klebsiella pneumoniae* strains included in the study^*a*^

MIC (µg/mL)	Zone (mm)																					
	7***	8***	9**	10**	12**	14**	15**	16*	17*	18*	19*	20*	22*	23*	24*	25*	26*	27*	29*	30*	32*	33*
0.03*																						
0.06*															1	1						
0.125*											1				1							
0.25*															1		1				1	
0.5*																		1	2			
1*									1					2			3	1	1		1	
2*					1	1	1			1			3			1		1				1
4*	1									1		1	1	2		3	2			1		
8**					1	1	2	1		1	1					1						
16***		1	1	1																		
32***																						

^*a*^ Zone diameters in millimeters are in the columns and minimum inhibitory concentrations (MIC) in µg/mL are in the rows. ***, **, and * indicate resistant, intermediate, and susceptible zone diameters or concentrations, respectively (CLSI criteria, 2022).

Nine (8.7%) isolates were susceptible only to FDC and resistant to all other antibiotics tested.

When the MIC value was interpreted using EUCAST breakpoints, 43% of the isolates were found to be resistant to cefiderocol (10). Similarly, categorical agreement with disk diffusion was 79% and 56% for susceptible and resistant categories, respectively. With EUCAST breakpoints, VME occurred in 19.2% and ME occurred in 11.5% (Tables 2 and 3).

**TABLE 2:** Categorical agreement between broth microdilution and disk diffusion for cefiderocol according to the CLSI and EUCAST breakpoints^*a*^

CLSI	Susceptible	Intermediate	Resistant
BMD	41	8	3
DD	37	4	1
CA (%)	90	50	33
**EUCAST**			
BMD	29	Not applicable	23
DD	23		13
CA (%)	79		56

^*a*^ BMD: broth microdilution, DD: disk diffusion, and CA: categorical agreement

**TABLE 3:** Error rates for disk diffusion of cefiderocol for CLSI and EUCAST breakpoints

	CLSI		EUCAST	
Very major errors	0	0.00%	10	19.20%
Major errors	1	1.90%	6	11.50%
Minor errors	5	9.60%	Not applicable	

### CST broth microdilution testing

CST BMD showed that 89% (92/103) isolates were intermediate (MIC ≤ 2 µg/mL) and 11 (11%) were resistant (≥4 µg/mL). For three of these 11 resistant isolates, discrepancies were observed with VITEK-2 MICs, two isolates were categorized as intermediate (MIC ≤ 2 µg/mL) by VITEK-2.

Four (3.8%) isolates were intermediate only to CST and resistant to all other antibiotics tested.

### CZA susceptibility testing

Disk diffusion testing for CZA showed 26.2% (27/103) isolates were susceptible, 1% (1/103) intermediate, and 72.8% (75/103) were resistant.

### Synergy testing between CZA and AT

Synergy testing was positive for 72% (74/103) of the isolates. Resistance to both CZA and AT was observed when tested individually in 80% (59/74) of these isolates. Synergy was present in 70% (14/20) isolates, which were non-susceptible (I, R) to FDC. Among the five isolates resistant (R) to FDC, only one showed synergy between CZA and AT.

## DISCUSSION

The national AMR surveillance network in India has shown a susceptibility of only 47% against meropenem for *K. pneumoniae* (6). This seriously limits the treatment options, necessitating the use of last-resort antibiotics.

FDC is an FDA-approved, parenteral, siderophore cephalosporin antibiotic, which acts by interfering with iron uptake by the microorganisms (11). The structure combines a catechol moiety with a cephalosporin core, giving it enhanced stability against many β-lactamases including MBL (12). It is recommended for the treatment of carbapenem-resistant Enterobacterales in regions with a high prevalence of MBL. Susceptibility testing of FDC by BMD requires the use of iron-deficient media, as iron interferes with the activity of this drug. This can be technically challenging, lowering the reliability and reproducibility of in-house testing. However, disk diffusion is performed without any additional requirements and is shown to be reliable (13).

CST belongs to the polymyxin class of antibiotics. The target serum concentrations are as high as 2 µg/mL for it to be effective, which is not achieved in more than 50% of the patients and results in nephrotoxicity (14). The susceptibility testing of CST shows low reproducibility and problems in interpretation due to heteroresistance. Disk diffusion, gradient testing, or semi-automated commercial testing panels (e.g., VITEK-2) are not recommended for testing this drug. The only recommended method is broth microdilution, which is not widely available, especially in resource-limited settings (15). Semi-automated methods such as VITEK-2 are used in such settings for performing susceptibility of CST. Khurana et al. found 10% VMEs with VITEK-2 (16). In our study, we also observed discrepancies between BMD and VITEK-2 for CST susceptibility, with lower VMEs at 1.9%. CLSI has also removed the category “susceptible” for this drug, keeping only “intermediate” and “resistant” categories for reporting (7). In our study, 89% of the isolates were intermediate to CST. This is in line with the findings of Amladi et al. and Manohar et al. (17, 18).

Ceftazidime-avibactam is a novel drug, which is shown to be effective against carbapenem-resistant Enterobacterales, except those producing MBLs (19). Since India has reported a high prevalence of MBLs (20), this drug alone is not very effective but can be used in combination with aztreonam (2). Low susceptibility was observed in our study, with only 26% of isolates being susceptible to CZA. Susceptibility testing is easily performed with routine disk diffusion. However, there are no guidelines for synergy testing between CZA and AT. A study by Sahu et al. (9) found that more than 80% of the isolates showed synergy for CZA/ AT and 96% of these harbored *bla*_NDM-1_. In comparison, synergy testing was positive in 72% of our isolates.

Our study shows less susceptibility (MIC_90_ of 8 µg/mL) than the SIDERO-WT study (MIC_90_ of 4 µg/mL) for carbapenem-nonsusceptible Enterobacterales (5). However, the study represented isolates majorly obtained from Europe and North America, with only 8% of isolates originating from Asia. Similarly, cefiderocol susceptibility performed on isolates collected during the SENTRY study showed MIC_90_ of 4 µg/mL for CRE, with CRE constituting only 2.1% of the study isolates (21). In another study from China, among the 105 carbapenem-resistant *K. pneumoniae*, 100% were susceptible to FDC. However, only eight isolates harbored *bla*_NDM-1_, remaining harbored *bla*_KPC_. Isolates harboring *bla*_NDM-1_ were found to have higher MICs for FDC in this study (22). Co-production of multiple carbapenemases could contribute to resistance as discussed by Kohira et al. (23). Indian isolates often harbor more than one carbapenemase gene (20, 24). This might have contributed to lower susceptibility in our study than that in the Western literature. Furthermore, resistance to CZA might contribute to reduced susceptibility to FDC, as noted by Bianco et al. (25).

In our study, there was a wide variation between susceptibility rates when applying CLSI and EUCAST breakpoints for FDC. A total of 80% and 56% of the isolates were susceptible to FDC when CLSI and EUCAST breakpoints were used, respectively. These findings are in line with Morris et al. (26). Disk diffusion is a convenient alternative to MIC testing for FDC, since BMD testing for the same has many technical challenges (27). Matuschek et al. have validated disk diffusion as a reliable and robust alternative to BMD (13). However, in our study, categorical agreement varied widely between the CLSI (CA = 80%) and EUCAST (CA = 69%) breakpoints. Error rates also varied widely with CLSI breakpoints giving less error rates as compared to the EUCAST criteria. This variation is also observed by Morris et al. (26) and Bonnin et al. (28). Although the methodology is different and the breakpoints are not interchangeable, it clearly indicates wide differences in interpretation, which might arise if one shifts from one methodology to the other. Hence, it can be argued that cefiderocol breakpoints need to be investigated further for harmonization, keeping in view the clinical response and PK-PD parameters, especially in areas with a high burden of carbapenem resistance.

Among other treatment options for MER-R *K. pneumoniae*, tigecycline was not compared in the study, as more than 70% of our isolates were from blood, respiratory system, or urine; all these are the sites where tigecycline is not effective. Fosfomycin was not compared as only 20% of isolates were recovered from urine. Molecular characterization for identifying the mechanism of carbapenem resistance of all the study isolates could not be performed, which is the limitation of the study.

### Conclusion

FDC exhibited reasonable *in vitro* activity against meropenem-resistant *K. pneumoniae* in our study isolates. Larger clinico-microbiological studies should be performed for assessing the clinical efficacy of this antibiotic in this region with a high prevalence of carbapenem resistance among Gram-negative organisms.
